# Supplementary material for: Global energy spectrum of the general oceanic circulation
Source: Nat Commun. 2022 Sep 9;13:5314. doi: 10.1038/s41467-022-33031-3 (PMC9463453; doi:10.1038/s41467-022-33031-3)
Supplement: Supplementary file 1 — Supplementary Information [file 41467_2022_33031_MOESM1_ESM.pdf]

Global Energy Spectrum of the General Oceanic Circulation

Supplementary Material

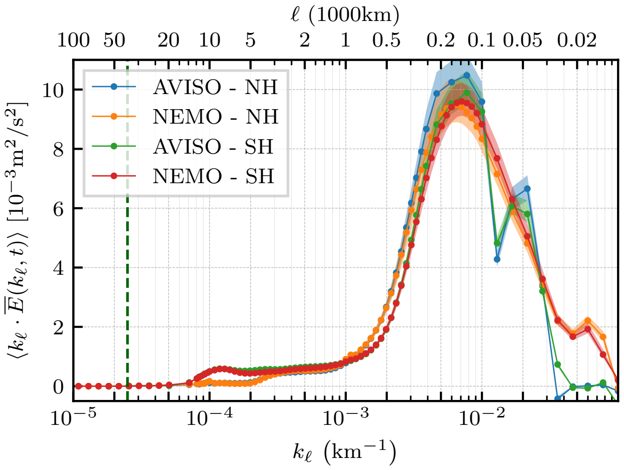

Fig. S1: Variance-preserving Spectra Same layout as upper panel of Fig. 2, but plotting the so-called variance-preserving spectra obtained by multiplying the spectra in Fig. 2 by k_ℓ.

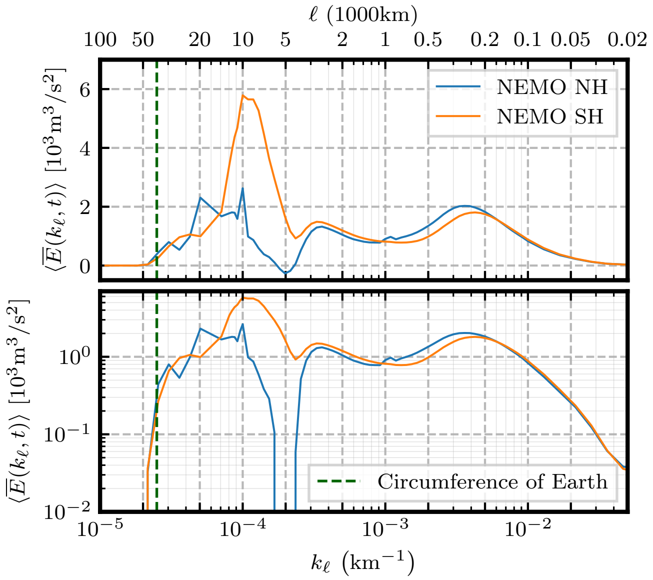

Fig. S2: Spectra without Hemisphere Reflections Same layout as Fig. 2, but without hemisphere reflections. That is, NH and SH spectra are extracted from a single global coarse-graining. Note that the NH spectra has a large-scale peak near $\ell = 20 \times 10^3$ km, which is not observed using reflected hemispheres. Produced using the same four-year period of NEMO data, sub-sampled to every fourth day.

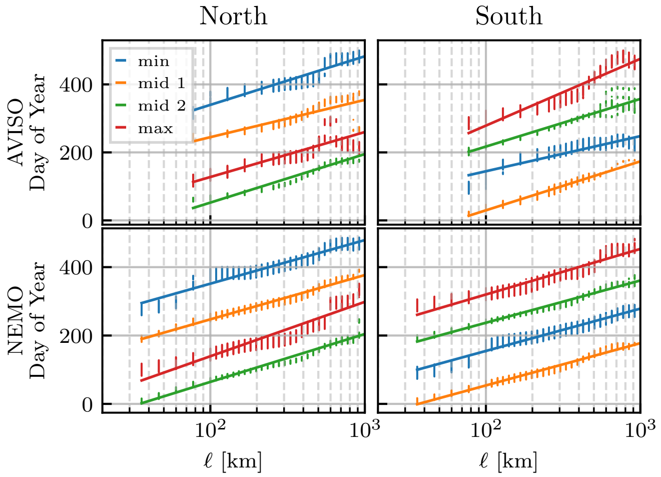

Fig. S3: Seasonality Regression Datapoints Extracted data points and regression fits for the seasonal drift exhibited in Fig. 3. Panel layout ordering is identical to Fig. 3. Dots (which appear as vertical bars due to their proximity) show the extracted k_ℓ-time points, and the lines show the corresponding regression fit. In the legend, 'min' means the lowest 10% z-score, 'mid' the two middle-most (or zero-crossings) 10% groups, and 'max' the highest 10%.
